# Addressing therapist concerns about religion in cognitive behavioural therapy: why, when, and how?

**DOI:** 10.1017/S1754470X26100646

**Published:** 2026-06-11

**Authors:** Hibah Hassan, Katherine E. Wakelin, Graham R. Thew

**Affiliations:** 1 Oxford Health NHS Foundation Trust, UK; 2 Hampshire and Isle of Wight Healthcare NHS Foundation Trust, UK; 3 Department of Psychology, University of Southampton, Southampton, UK; 4 Department of Experimental Psychology, University of Oxford, UK

**Keywords:** access, alliance, barriers, religion, spirituality

## Abstract

Despite religion being an important part of some patients’ identity and value systems, therapists report finding it challenging to discuss religion in therapy. Avoiding religion for these patients can have detrimental effects on the therapeutic alliance, treatment outcomes, and parity of access for minoritised groups. Therefore, this clinical guidance paper aims to support therapists to bring religion into the therapy room by addressing six key concerns: (1) difficulties raising the topic, (2) ‘getting it wrong’ when discussing religion, (3) therapist and patient differences, (4) managing negative or ambivalent beliefs about religion, (5) perceptions of insufficient knowledge, and (6) a lack of time for meaningful discussions. These barriers are explored by drawing on empirical evidence, clinical experience, and illustrative case examples. The paper aims to provide suggestions and next steps for how therapists can reflect on and address these concerns, aiming to enhance confidence and competence in integrating religion into CBT.

## Introduction

When working holistically in therapy, it is important that therapists attend to all aspects of a patient’s identity and value systems that are held as important, including but not limited to, race, culture, and religion (Beck, 2016). Religious beliefs in particular are often overlooked in clinical therapeutic settings (Frazier and Hansen, 2009; Hodge, 2013; Hathaway *et al*., 2004). This has been observed despite the extent to which someone identifies as religious being a variable of interest when considering disparity in access to mental health services. In the United Kingdom, religious individuals are less likely to access talking therapy than those who do not identify as religious (Office for National Statistics, 2022). Furthermore, individuals from certain religious groups, such as Muslims, have poorer outcomes compared with patients who do not identify as religious (NHS England, 2024; Shafan-Azhar *et al*., 2025). Therefore, it is important to understand the barriers that religious individuals face to accessing therapy, as well as the barriers to achieving positive outcomes when in treatment.

Research suggests that perceptions of therapy are impacted by whether religious individuals believe that their therapist will incorporate religion into treatment, and that patients prefer a therapist who is able to discuss religion with them and incorporate it into treatment (Ayub and Macaulay, 2023; Oxhandler *et al*., 2021; Weatherhead and Daiches, 2010). For example, a recent video-vignette study found that when British Muslims were shown a therapist who explicitly acknowledged religion as part of therapy, they rated cognitive behavioural therapy (CBT) as more credible and had higher expectations of improvement, compared with when the same therapist described attending to their general values (Hassan *et al*., 2024). This suggests that when clinicians offer to discuss religion, patients may have more positive expectations of therapy. As said by Pargament (2011), ‘*when people walk into the therapist*’*s office, they don*’*t leave their spirituality behind in the waiting room*’.

Having open discussions about religion can support rapport building and enhance therapeutic alliance by demonstrating an openness to a patient’s view of life (Blair, 2015; Jacobs, 2010; Magaldi-Dopman *et al*., 2011). Discussing religion also allows for a deeper and fuller understanding of a patient’s reality, experiences, and coping mechanisms (Pargament, 2011), enabling a richer formulation to base treatment on. Moreover, religious resources may be mobilised to aid the therapeutic process and be a key ingredient to successful treatment (Wakelin and El-Leithy, 2025). Conversely, avoiding or failing to discuss religious beliefs can have negative consequences including ruptures in the therapeutic relationship, premature termination, and poor therapy outcomes (Hodge, 2013; Owen *et al*., 2014). This may be especially detrimental when working with highly religious patients (Judd, 2019), who may demonstrate a greater sensitivity to how their therapist attends to religion.

Growing research highlights how religion-adapted psychotherapy, including CBT, is at least as effective, if not more, than conventional psychotherapy for mental health difficulties (Anderson *et al*., 2015; de Abreu Costa and Moreira-Almeida, 2022; Gonçalves *et al*., 2015; Munawar *et al*., 2023). Commonly, the adaptations include the integration of religious content into techniques such as cognitive restructuring and psychoeducation, the use of religious activities to support behavioural activation and use of positive coping strategies, and motivating patients to act according to their religious values (de Abreu Costa and Moreira-Almeida, 2022). Religion-adapted psychotherapy is indicated to be more effective the stronger someone’s religious identity is (Wade *et al*., 2007). However, in public health services with limited funding, interventions that do not require specialist training or access to additional protocols, and those that can be delivered by both religious and non-religious therapists routinely, are desirable (see Wakelin and El-Leithy, 2025).

In the absence of specific religion-adapted psychotherapy, religious patients may find it difficult to raise the topic of religion themselves (Gockel, 2011; Knox *et al*., 2005). However, research suggests that therapists may also struggle to do this for a variety of reasons, including their own discomfort around the topic, wanting to protect the therapeutic alliance, and holding beliefs that working with religion is outside of their area of expertise (Crossley and Salter, 2005; Knox *et al*., 2005; Rose *et al*., 2001; Magaldi and Trub, 2018; Mayers *et al*., 2007; Naz *et al*., 2019). The consequence of therapist ambivalence is likely to further marginalise religious patients who may feel that therapy is not a space where they are able to bring a central part of their identity.

It has been recommended that clinicians take a proactive approach to discussing religion during therapy, aiming to create an environment in which patients feel able to speak about their religious beliefs and identity (Hassan *et al*., 2024). Given the documented difficulties and discomfort therapists may experience, this paper aims to summarise common therapist concerns around discussing religion in CBT, and offer potential routes forward. This is done by drawing on empirical evidence, clinical experience, and a series of illustrative case examples. The case examples are, generated from the authors’ clinical practice. The paper considers six key therapist concerns which may be relatable for therapists who are religious, as well as those who are not religious: (1) difficulties raising the topic of religion, (2) ‘getting it wrong’ when discussing religious topics, (3) highlighting differences between you and your patient, (4) managing your own or others’ negative or ambivalent beliefs about religion, (5) feeling you have insufficient knowledge to discuss religion, and (6) a lack of time for meaningful discussions.

Although the focus of the paper is on CBT, it is anticipated that most of the principles and strategies are transferable across therapeutic modalities, and will be thought-provoking for a range of therapists. Furthermore, this paper predominantly uses the term religion rather than spirituality. This is because religion is a way in which some people express their spirituality, but not all spiritual individuals are religious. The case examples draw primarily from the more widely practised religions however, the principles and ideas discussed aim to be transferable and support inclusivity across all religions, spiritual beliefs and experiences, including minoritised or diasporic faiths and traditions.

### 1 Raising the topic

Concern: Raising the topic of religion may elicit discomfort in therapists, particularly in those who may not be accustomed to discussing the topic professionally or otherwise. Example therapist beliefs and questions here may include:

- What if it’s not relevant?
- The patient might not want to talk about it
- What if they are offended that I’m enquiring about a personal matter?
- How do I bring it up in a relaxed, non-judgemental way?

Points to consider: Avoiding discussion of religion in therapy may be easy to do in many cases. However, as with other avoidance strategies, there can be unintended negative consequences. For the patient it could mean they do not feel understood or connected with (Hassan *et al*., 2025), or that their therapist does not really appreciate what might be important to them. In some cases avoiding religion may also be perceived as a microaggression. Patients may therefore feel less close to their therapist and have doubts about whether the treatment will be able to help them (Wade *et al*., 2007). For the therapist, this avoidance may make it harder to fully understand the patient’s perspectives and how they view the world. Therapists are also more likely to miss out on important beliefs or assumptions that patients hold about their difficulties and how to address them, or possible barriers and facilitators to successful treatment. Lastly, of course therapists avoiding religion do not get to discover what actually happens if they raise the topic of religion and whether the specific concerns they hold are valid. As CBT practitioners, this proposes a potential opportunity for us to ‘take our own advice’ and try this out as a behavioural experiment within our clinical work where appropriate.

We suggest that in the course of assessment or initial therapy sessions where there is a focus on understanding the patient, broad questions are presented with genuine curiosity. For example, ‘I am mindful that for some people religion can be a significant part of their identity. As we are getting to know each other, I am wondering if religion is an important part of your life?’. Such questions enable a patient to say ‘no’ so the conversation can move elsewhere or only respond briefly if they would prefer not to disclose their religious identity at that time. Closed questions such as ‘Do you follow a particular religion or belief system?’ may be less helpful if patients no longer identify with a religious belief and may not communicate the therapist’s interest in exploring how faith may be relevant more broadly to their family or to their earlier experiences.

Some practitioners use a list or diagram of all the protected characteristics from the Equality Act (2010), or models such as the Social Graces (Burnham, 2018) to invite the patient to speak about any areas that are important to them and their identity, or may incorporate religious beliefs into genograms. For some patients, there may be thematic overlaps in areas of religion and culture, and it is important that therapists take the time to understand and distinguish between the impact of religion and culture and to avoid the conflation of the two. If using such approaches, it is recommended to do this routinely with patients, not just with those where religious beliefs may be indicated via clinical notes, verbal discussion, or visual cues.

If the patient does identify as religious, helpful follow-up questions to explore this could include:

- ‘I noticed that on your assessment form you mentioned identifying as religious, is it okay if we talk about that?’
- ‘Do you observe any religious practices, such as attending a place of worship or regular prayers?’
- ‘How does faith impact your daily life?’
- ‘What is it like to be part of your religious community? Do you receive any support from the community?’
- ‘Have you sought support from religious leaders for your mental health difficulties? What has your experience of this been?’
- ‘Was religion important in your family or upbringing?’
- ‘How does your faith help you in times of difficulty?’
- ‘What does your faith say about people getting help for difficulties with their mental health?’

#### Case example 1

*Shamshad, a 31-year-old, attends his assessment appointment with therapist, Layla. Layla wonders from Shamshad*’*s name whether he might be from an ethnic minority background, but that the religion section on his pre-assessment form has been left blank. Layla wonders whether religion is unimportant to Shamshad and at assessment enquires about this, asking*, ‘*I noticed that your assessment form did not mention any religious or spiritual beliefs, it*’*s something we ask everyone about, and I wondered if it was relevant to you at all?*’. *Shamshad explains that although religion is important to him personally, there has been significant tension and upheaval in his family along the lines of religious difference, and he therefore chooses not to explicitly name his religious identity on paperwork. Layla acknowledges this and says it sounds like it would be important for them to think about the difficulties within the family, and they agree to discuss religion in this context*.

### 2 Getting it wrong

Concern: This group of concerns relates to the general idea that raising the topic of religion could go wrong and generate awkwardness or embarrassment for the therapist and/or patient. Example therapist beliefs and questions may include:

- I will say something wrong and offend them
- They will see I don’t know what I’m talking about
- It will seem like I’m making assumptions about them based on their background or appearance
- How will the relationship recover if I make a mistake about something so important to them?

Points to consider: Taking a cognitive behavioural perspective on these fears, they carry the underlying meaning that as therapists, we might come across as intrusive, ignorant, offensive, or generally incompetent. This of course would have important consequences for the treatment, jeopardising the safe and supportive environment and strong therapeutic alliances we aim to develop. Therefore, there are a range of potential negative consequences, both for our own self-belief, as therapists and as humans, and for the success of the treatment if we make mistakes when discussing religion or spirituality. As a result, it is highly understandable that many therapists engage in behavioural avoidance strategies to avoid ‘getting it wrong’, either in full (not discussing the topic at all), or in part (only discussing if the patient mentions it).

Although ‘getting it wrong’ is something most practitioners would prefer to avoid, we are all human, and it is therefore normal that various misunderstandings, mistakes, or assumptions might occur during therapy about religion, or otherwise. It has been suggested that failing to ask about a patient’s broader background due to fears about getting it wrong is still getting it wrong, just in a different way (Griffiths *et al*., 2024). It may therefore be helpful to embrace the idea that we might make mistakes at times and consider instead how we handle this. Although therapists should take responsibility to familiarise themselves with the key ideas and practices of different religions, we cannot know everything, and certainly we cannot assume that we know the nuances of religion for a given patient. It may be helpful for the therapist to name the patient as the expert on their own experiences and the therapist the expert on CBT, for them to work collaboratively together would mean to share and ask questions about each other’s expertise. In particular, therapists should therefore adopt a position of curiosity about and explore the patient’s *experience* of the religion, regardless of their knowledge or own familiarity with the religion. This may be particularly important if a therapist and patient share a religious background, where assumed similarity may become a hindrance to a thorough understanding of the patient.

Phrases such as ‘Would it be accurate to say … ?’, or ‘Could you help me understand … ?’ can be helpful when exploring religious ideas that are less familiar (see also Sensoy and DiAngelo, 2014). Therapists who have taken the time to do some background reading may wish to name the possibility that they will make mistakes while demonstrating their proactive approach towards working with religion, ‘I have done some research on your religion but I’m likely to get things wrong at times, I hope that when this happens, we can work through it together’. If something does ‘go wrong’, we recommend apologising and owning the mistake. For example, ‘Sorry I think I got that wrong’, or ‘It looks like I made an assumption there … ’. Then, if possible, exploring the patient’s experience further, for example, ‘I wonder what went through your mind then when I got that wrong?’. This may therefore be an opportunity to model the ‘okay-ness’ of making mistakes and the subsequent process of acknowledgement and repair, which when successful, can have positive impacts on the therapeutic relationship.

#### Case example 2

*Sangita is attending her first session with her therapist Arthur. Sangita rushes in late to the session and explains apologetically that she was held up at the temple. Arthur, wanting to take the opportunity to show his openness to discussing faith, comments* ‘*Oh, I walk past the Hindu temple on my way to work!*’. *Sangita explains that she is a Sikh, not a Hindu. Arthur notices a sense of embarrassment and feels himself going red. He decides not to engage in behavioural avoidance and instead owns his mistake, saying* ‘*Gosh, I*’*m sorry, I got the wrong religion there, didn*’*t I? I*’*m not very familiar with Sikhism but I*’*d really like to learn more about it and how it is relevant to you*’. *Sangita smiles warily at Arthur. Noticing this, he asks what went through Sangita*’*s mind. She explains that she*’*s tired of ignorant people lumping all religious individuals together into one group. From this understanding Arthur is able to explore experiences of discrimination that Sangita faces. He brings his feelings about being perceived as ignorant to supervision*.

### 3 Highlighting therapist–patient differences

Concern: This group of concerns centres on the idea that talking about religion might highlight therapist–patient differences that could hinder the therapeutic relationship and/or have a negative impact on clinical outcomes. For some patient and therapist dyads, differences may be visible, for example, if the therapist wears a kippah or a turban; for others naming difference may be a more deliberate choice. Example therapist beliefs and questions may include:

- The patient might think I can’t help them if I don’t share their beliefs
- They won’t want to work with me if I draw attention to our religious differences
- What if they ask me if I believe in God or whether I am religious?
- It’s better to focus on our shared goals rather than our differences
- We’re too different to work together successfully

Points to consider: Many of these concerns rest on the assumption that therapy is more likely to be successful when the therapist and patient share similar religious beliefs. However, studies evaluating religion-adapted therapy protocols have found that the therapist’s own religiosity did not influence clinical outcomes (Anderson *et al*., 2015; de Abreu Costa and Moreira-Almeida, 2022; Lim *et al*., 2014).

It is important that therapists respond to questions about differences in faith in a way that acknowledges the importance of faith to the patient. Negative or neutral responses to questions about therapist religion such as ‘I wonder why my beliefs are important to you?’ or ‘I consider all religions to be equally important’ may feel invalidating, or leave patients feeling like their therapist will not understand the personal weight and importance of their belief system (Judd, 2019).

Responses to such questions may or may not include self-disclosure, which when used with deliberation and in service of the patient, can strengthen the therapeutic relationship (Hanson, 2005; Goldfried *et al*., 2003). Phiri *et al*. (2019) suggest that some patients appear to test whether therapists are genuine, trustworthy, and able to connect with them on a human level by initiating self-disclosure, but that it is not the content of the response they are testing, rather whether the therapist responds sensitively and is attuned to what is important to the patient. Therapists should therefore be prepared to address moments in which patients may be probing for self-disclosure (Magaldi and Trub, 2018).

Below are some suggested phrases to do this, with and without self-disclosure:

- ‘Before I answer that question, I want to think with you about how it might feel or what impact it might have on our work if I share that I do not identify with a religion … and how might it feel if I were to share that I do identify with a religion … Now, would you still like to know the answer?’
- ‘It’s understandable that you might be curious about that. Before I answer, I wonder how it might feel or what impact it might have on our work if I answer one way or another?’
- ‘I do not hold religious beliefs myself, but I can hear that this is central to your life and your decisions, so it’s important we think about it together’
- ‘If it’s important to you then it’s important to me and our work together’
- ‘I’m hoping that I can appreciate and understand how central your faith is to you even though my beliefs may be different to yours’

Once a level of religious alignment or difference has been identified and acknowledged, it may then be helpful to explore the patient’s individual beliefs about this, to elicit any concerns and anticipate any potential barriers or facilitators to therapy, for example:

- ‘How might you view me and our work together differently if we hold quite different beliefs?’
- ‘Do you have any concerns or questions about how we might work together if we hold quite similar beliefs?’
- ‘Might there be any benefits to this difference/similarity?’

#### Case example 3

*Janine is a 73-year-old Christian woman who presents with low mood. Her therapist, Lauren, is 26 years old. At their initial meeting, Janine comments on their great age difference and wonders out loud how Lauren, with less life experience than her, would be able to help her. Lauren acknowledges Janine*’*s concerns, explains that she is fully qualified, and hopes they can combine her training and theoretical knowledge together with Janine*’*s life experience and wisdom. During the assessment Janine shares that attending church weekly brings her great comfort through connection to God and brings structure to her week. She asks Lauren*, ‘*Do you believe in God?*’. *Not wanting to highlight the difference between her beliefs and Janine*’*s, Lauren responds with* ‘*I wonder why my beliefs are important to you?*’. *Janine is left feeling like Lauren does not understand the personal importance of her faith and comments that she is not sure the pair can work together because of differences between them. Lauren reflects on the rupture in supervision the following week. With her supervisor, Lauren considers the different perspectives they both hold, and the assumptions her and Janine may have made*.

### 4 Managing negative or ambivalent beliefs

Concern: Therapists often report discomfort at considering religion in therapeutic work (Rosmarin *et al*., 2013), sometimes due to their own negative or ambivalent beliefs. Personal negative or ambivalent beliefs about religion may be something therapists recognise in themselves, or are aware of more externally in peers, supervisees or team cultures. Example negative or ambivalent beliefs therapists could hold may be:

- Religion is a negative influence
- A patient’s religious beliefs are maintaining their distress
- Other religions get things wrong compared with my own religion
- Religion is irrelevant to therapy
- Spirituality is private
- A patient’s religion is not my business

Of note, 39% of religious patients in psychotherapy indicate that at least one religious microaggression occurred within treatment (Trusty *et al*., 2022). This included the minimisation/ avoidance of religious issues or prioritisation of the therapist’s religious views. Therefore, even if a therapist is not actively negative towards religion, microaggressions can be expressed through therapist ambivalence. This is a concern because religious microaggressions are negatively associated with the therapeutic alliance and therapy outcomes (Trusty *et al*., 2022), meaning the more microaggressions patients reported, the lower the ratings for therapeutic alliance were. Therefore, therapists hold an ethical responsibility to identify and manage their personal views of religion to enable spiritually competent practice. Microaggressions may be more prevalent in the context of sociopolitical contexts and discrimination more broadly. An example of this is the rising levels of Islamophobia reported across Europe and the United States since the 2001 9/11 terrorist attacks (Ogan *et al*., 2014). This is something for the therapist to hold in mind and reflect on in relation to their own practice.

Points to consider: Encouragingly, spiritually competent therapy can be increased through clinician education and support (Rosmarin *et al*., 2013). Therefore, through self-reflection, a therapist can identify views they personally hold towards religion and consider how any ambivalence or negativity may impact their practice. Griffith and Griffith (2002) recommend that therapists strive to become aware of their own cynicisms and certainties regarding religion to develop an attitude of ‘wonder’ about the patient. Below are some suggested questions that a therapist may find useful to aid private self-reflection:

- What are my personal views and attitudes towards religion?
- How do I refer to or talk about religion with my friends, family, colleagues, and patients?
- What type of language do I use?
- What personal experiences have influenced my view of religion? How do I make sense of these experiences?
- Do I have an immediate/automatic response when someone talks about religion?
- How may my own personal experiences of religion be impacting how I interpret and respond to my patient’s religion?
- How can I separate my own experiences of and beliefs about religion from my patient’s?

Therapist curiosity is also developed and supported through supervision, where therapists can consider how their own belief systems and identities impact their practice with facilitated support. However, supervisors may also differ in their positioning towards and self-reflection on religion. Therefore, visual exercises such as genograms or Salient Circles can be useful prompts for facilitating both the supervisee and supervisor to reflect on their belief systems (Buchanan, 2020; Shellenberger *et al*., 2007). Such exercises may enable therapists to consider how multiple parts of identity, such as religion, age, migration patterns and ethnicity, intersect to create a unique viewpoint of the world (Butler, 2017; Collins, 2000). Below are some discussion starters that a supervisee and/or supervisor may find useful to begin discussions around religion within supervision, developed from Sensoy and DiAngelo (2014):

- ‘I’m really nervous/scared/uncomfortable to say [X], but … ’
- ‘I’m not sure if this will make any sense, but … ’
- ‘Can you help me understand whether what I’m thinking right now might be problematic?’
- ‘I am having a “yeah, but” moment. Can you help me work through it?’
- ‘How does [X] affect relationships between [Y] and [Z]?’
- ‘This perspective is new to me, but I’m wondering if it is accurate to say that … ?’

Finally, it is beneficial for therapists to remind themselves of the evidence base of religious adapted CBT. Therapy seeks to build on a patient’s strengths and resources, and spiritual beliefs, practices and communities are often a significant source of strength for many patients (Van Wormer and Davis, 2013). Religion has the potential to increase positive emotions, offer a sense of purpose and give meaning to adversity (Koenig, 2012). This counters beliefs that religion may be dangerous or negative, or that it is ‘irrelevant’ or too ‘private’ for therapy. Therefore, therapists are encouraged to hold ‘curious wonder’ for each individual patient’s relationship with religion.

#### Case example 4

*James is a white British, male therapist working with Amina, a British Pakistani Muslim woman struggling with low mood. Within supervision James shares that he is finding it hard to build a rapport with Amina and engage her in behavioural activation work. With support from his supervisor, James reflects that he grew up within a Christian schooling system and attended church on occasion. However, he never felt a connection to God. As an adult, James does not hold any negative feelings towards religion, but feels it to be largely disconnected to his life today and would not describe himself as religious. James and his supervisor identified how James*’ *ambivalence towards religion has naturally prevented him from being curious about Amina*’*s Muslim faith. Following conversations in supervision, James says to Amina*, ‘*I notice you mentioned previously being part of a mosque, I*’*m wondering if this is something that is still important to you?*’. *This leads Amina to share that when her mood is low, she struggles to shower, which prevents her from being able to pray and attend the mosque. This is due to Islamic teachings around prayer and ritual cleanliness. Recognising Amina*’*s goal to re-engage in her spiritual life gave activity scheduling personal significance, and their remaining sessions were able to prioritise working on her physical and ritual cleanliness to allow her to re-engage in prayer*.

### 5 Therapist lack of knowledge

Concern: Another commonly held concern by therapists is the potential to be placed in a position of guidance on religious matters, or a sense that the issue a patient is bringing is a religious issue rather than a mental-health concern. Therapists may feel ill-equipped to provide such guidance or address these issues due to a lack of familiarity with the specific religious context but still feel a sense of responsibility to guide patients in a way that is in-keeping with their faith beliefs. Therapists may be aware that insensitivity to a patient’s beliefs could be detrimental to the therapeutic alliance or the patient’s ability to engage in therapy, thereby placing them under greater pressure to ‘get it right’. Example beliefs may include:

- This is outside the scope of therapy
- This is more about religion than it is about mental health
- What if I suggest something that goes against their religious beliefs?
- I am not a religious expert
- How do I know what is allowed or appropriate?

In the treatment of obsessive compulsive disorder (OCD), some patients may experience fears about engaging in exposure exercises that may include deliberately repeating behaviours that they believe violate their religious rules. For further guidance, see Abramowitz (2001) and Huppert and Siev (2010).

Points to consider: If therapists are questioning where the boundary is between what CBT can intervene with, and what is personal to someone’s religion, we suggest this distinction is guided by the patient’s formulation and reflecting on the maintenance cycles of distress in supervision. It may also be useful to clarify with the patient what their goals for therapy are, and what information is necessary to attain these goals. Carefully formulating the cause of distress and how it may manifest is important to help both patient and therapist see what information or skills are needed to move forward. A joint problem-solving approach may be useful here in identifying where religious information can be gathered from. This may serve to strengthen the therapeutic alliance if the patient feels that the therapist is willing to put the time and effort into valuing their religious beliefs.

If it appears that the patient has questions about their faith that the therapist is not placed to answer, it may be beneficial to seek guidance from religious leaders in treatment, as suggested by the National Institute for Health and Clinical Excellence (2005). As there are many religions, and more denominations within each religion, even therapists with good knowledge of religion may never know ‘enough’. Therefore, it may be useful to consult with a religious leader outside of the patient’s personal network to reduce fears of confidentiality being broken. In the United Kingdom, this may involve reaching out to chaplains who are available in NHS trusts. Wakelin and El-Leithy (2025) discuss practical considerations for identifying and seeking advice from religious experts using interviews and surveys in cognitive therapy for post-traumatic stress disorder, which are generalisable across CBT for a range of mental health difficulties.

Below are some discussion starters therapists may find helpful:

- ‘I want to make sure that we are supportive of your religion, because I know that is important to you … Could you help me understand what the rules or boundaries are in a religious context?’
- ‘Perhaps we can put together my therapy skills with your knowledge of your faith and work together to make sure we do things the way you believe they should be done?’
- ‘This dilemma seems to be more about religion than about your difficulty with X, I wonder if there is a religious figure that is better placed to help us understand it better?’
- ‘I feel like you are asking me whether something is allowed or not, but unfortunately, I do not have this information … Who do we know that might?’
- ‘I want to help you practise your religion as it was intended rather than out of fear … ’

#### Case example 5

*Isaac is a male Orthodox Jewish man who presents with OCD. He doubts the completeness of his faith and the strength of his relationship with God in his daily prayers and finds himself having to start over repeatedly. Having to repeat prayers is distressing for Isaac as he is not able to say each prayer the right number of times as commanded in the Jewish faith. Isaac has also been seeking reassurance excessively from his Rabbi. His therapist, Salim, a Christian man, holds knowledge of the importance of concentration and mindfulness during prayers but he is not familiar with the Jewish prayers. To assess the extent of Isaac*’*s repetition, Salim asks Isaac to say the prayer in the therapy session in front of him, and Isaac does so without any issue. Salim is confused about this and asks Isaac to repeat the prayers. Isaac looks uncomfortable with this suggestion and explains that repeating God*’*s name in vain is a sin in Jewish law. Salim worries that he is asking Isaac to commit a transgression and explains that the dilemma ahead of them is a religious question rather than an OCD question. Salim and Isaac decide together to invite Isaac*’*s Rabbi to the next session. Salim speaks to the Rabbi ahead of time, explaining the treatment approach and the seriousness of Isaac*’*s problem. The Rabbi explains that for the purposes of treatment, it would not be considered sinful for Isaac to take God*’*s name in vain. He also agrees with a compassionate response to remind Isaac that his reassurance seeking is likely perpetuating his doubts*.

### 6 Lack of time

Concern: Due to the limited nature of many services delivering CBT interventions, it is understandable that therapists may feel there is limited scope in sessions to discuss religion meaningfully. Therapists may also hold concerns that:

- I don’t have enough time to be able to discuss religion with my patient
- If I don’t ask about religion initially, then it’s awkward to enquire later
- Discussions of religion could be lengthy and dominate the sessions
- If we don’t have time to discuss religion in depth, then there is little value in bringing it up

Points to consider: Beck (2016) provides a comprehensive guide for how, and when, to explore issues of spirituality, religion and culture in CBT. Therapists are encouraged to become comfortable routinely facilitating initial discussions about these issues with all patients in early sessions, after initial rapport has been established, as discussed in the earlier section on ‘raising the topic’ (Beck, 2016; Wakelin and El-Leithy, 2025). These discussions need only be brief if time is limited.

However, if religion holds significance for a patient, they are likely to mention or refer to it early in therapy, providing a natural opportunity for the therapist to ask a few further follow-up questions in a seamless manner (Wakelin and El-Leithy, 2025). Griffith and Griffith (2002) recommend that therapists are continually ‘listening out for the sacred’. This means listening out for language that indicates spirituality, for example ‘I felt so at peace’, ‘I deserve this punishment’ or ‘It’s in God’s hands now’. Therapists can then ask questions when patients use such phrases to respectfully query further, without taking up much time in therapy (Griffith and Griffith, 2002).

Another way to open the discussion early in therapy may be when assessing someone’s risk. Religion is frequently reported as a protective factor against suicide (Burshtein *et al*., 2016). Therefore, questions such as ‘Some people hold religious or spiritual beliefs that keep them going in times of crisis or difficulty … I am wondering if this is relevant to you at all?’ may be helpful. When mapping out a CBT formulation with a patient, it is encouraged to map out their strengths and resources, and consider their cultural influences (Naeem, 2019). This conversation provides another natural opportunity to check in if a patient is part of any communities, including religious, that are important to them, or hold any religious beliefs that are significant to them in terms of supporting them through adversity.

If religion is not acknowledged at the beginning of therapy, ample opportunities are present later; for example, when reviewing and building someone’s social support, or during activity scheduling, or behavioural experiments. In addition, working with cognitive themes of guilt, shame and moral injury and ‘reclaiming life’ work in cognitive therapy for PTSD provide further opportunities (Wakelin and El-Leithy, 2025).

Overall, where there are time pressures, therapists may feel the urge to rush or avoid discussions around religion. However, investing a little extra time to explore such viewpoints often enhances the collaborative alliance, and can make therapy more efficient and effective (Naz *et al*., 2019). Below are some possible questions therapists may want to consider in supervision or through self-reflection:

- How might asking one question about a patient’s religion have a positive impact on treatment?
- How much time would asking about religion when formulating take up practically?
- What concerns about the time involved do I hold that could be tested out in a behavioural experiment of my own?

#### Case example 6

*Michael is an atheist therapist working in a busy psychological therapy service with Cleo, a white British woman, presenting with PTSD related to child sexual abuse. Michael was aware from Cleo*’*s notes that she had converted to Islam in her adult life. However, they did not initially discuss religion in much detail as Michael felt they needed to make the most of therapy time and get going on memory-focused CT-PTSD therapy techniques early on. However, when they were restructuring cognitions of being objectified and worthless in a sexual assault hotspot, Michael said* ‘*I am mindful you mentioned you are a Muslim* … *I am wondering if there is anything the Quran teaches about your value and worth we could draw on here when updating these thoughts around worthlessness?*’. *Cleo was able to link teachings from the Quran about the value of every being, Allah*’*s omnipotence and justice in an afterlife into her updates to reduce her sense of shame, powerlessness and injustice in the assault memory. This gave the updates a religious meaning that would not have been achieved otherwise, resulting in more efficient and effective processing of the memory. Cleo addressed Michael*’*s concern about making the most of time in therapy by giving feedback that incorporating religion in therapy* ‘*has helped me so much, because it*’*s not something you gave me, it*’*s something that I already had in my life as an authority and follow* … *So then to bring in those parts* [from the Quran] *was incredibly helpful because I couldn*’*t argue with it*’.

## Conclusion

This paper aimed to consider the ‘why, when, and how’ of discussing religion in CBT, with reference to common concerns that therapists might hold about doing this (summarised in Table 1). In terms of the ‘why’ question, we have considered the evidence demonstrating how the integration of religion into therapy can be both meaningful and improve the experience of therapy for patients who identify as religious. It is likely to be beneficial through a combination of direct (e.g. improving the identification of key meanings and maintenance cycles) and indirect mechanisms (e.g. improving the quality of the therapeutic relationship, facilitating comfort in the relationship and motivation for change). Beyond this, there may also be clinical benefits in terms of improving the credibility of, and expectations for, treatment. Importantly, by developing our skills and confidence by discussing religious topics in CBT, we can hopefully begin to address the inequalities seen for patients from some religious backgrounds regarding treatment access and outcomes. For the ‘when’ question, we have offered guidance and suggestions for exploring religion at the initial assessment stage of treatment, as well as highlighting that there may be opportunities throughout therapy to do this, and that it is not too late to do this further into treatment. For the ‘how’ question, we have provided example questions, points for self-reflection or discussion in supervision or with colleagues, and clinical examples to offer practitioners possible routes forward. We hope that the information, ideas and strategies described might prove helpful to therapists to take forward and use in their practice.

We end this paper with a question from one of our patients who eloquently highlighted the need to attend to religion in therapy: ‘*I think it*’*s just asking, and coming from an open place* … *it*’*s something that*’*s very important to me, so why wouldn*’*t integrating it into my recovery be a good idea?*’.

## Figures and Tables

**Table 1:** Summary of possible therapist concerns about discussing religion in CBT

Therapist concern	Example beliefs and questions	Points to consider
**Raising the topic**	What if it’s not relevant? The patient might not want to talk about it What if they are offended that I’m enquiring about a personal matter? How do I bring it up in a relaxed non-judgemental way?	Use an open and curious approach Facilitate discussions using lists of protected characteristics or genograms Conduct your own behavioural experiments to try this out and test your concerns
**Getting it wrong**	I will say something wrong and offend them They will see I don’t know what I’m talking about It will seem like I’m making assumptions about them based on their background or appearance How will the relationship recover if I make a mistake about something so important to them?	Be mindful that avoiding the topic might still be ‘getting it wrong’, in a different way Develop familiarity with key ideas and practices of different religions Use tentative phrasings e.g. ‘would it be accurate to say … ’ Apologise and own any mistakes if they occur
**Highlighting therapist**-**patient differences**	The patient might think I can’t help them if I don’t share their beliefs They won’t want to work with me if I draw attention to our religious differences What if they ask me if I believe in God or whether I am religious? It’s better to focus on our shared goals rather than our differences We’re too different to work together successfully	Respond to questions in a way that acknowledges the importance of faith to the patient Be prepared to answer direct questions about religiosity Consider the value of self-disclosure in answers Explore how difference makes the patient feel about therapy
**Managing negative or ambivalent beliefs**	Religion is a negative influence Their religious beliefs are maintaining distress Religion is irrelevant to therapy Spirituality is private A patient’s religion is not my business	Consider self-reflection using suggested questions listed Use visual aids of cultural genograms or salient circles exercise as discussion starters in supervision Remind yourself of the evidence base of religious adapted CBT
**Therapist lack of knowledge**	This is outside the scope of therapy This is more about religion than it is about mental health What if I suggest something that goes against their religious beliefs? I am not a religious expert How do I know what is allowed or appropriate?	Ensure a clear understanding of the patient’s goals Adopt a joint problem-solving approach to attain missing information Seek guidance from religious leaders when appropriate
**Lack of time**	I don’t have enough time to be able to discuss religion with my patient If I don’t ask about religion initially, then it’s awkward to enquire later Discussions of religion could be lengthy and dominate the sessions If we don’t have time to discuss religion in depth, then there is little value in bringing it up	Facilitate initial brief discussions about religion in early sessions Listen out for references to religion throughout treatment and use these as natural points to follow up on Take opportunities to discuss religion when assessing risk, formulating strengths and coping, considering social support, values work, activity scheduling, and cognitive work

## Data Availability

Data availability is not applicable to this article as no new data were created or analysed in this paper.

## Key learning aims

(1) To support therapists to overcome commonly held concerns around discussing religion in therapy.
(2) To provide practical guidance, tips, and suggestions for how therapists can discuss religion with patients.
(3) To help therapists to take responsibility for bringing religion into the therapy room where it is relevant.

## Key practice points

(1) Therapists should take a proactive and curious approach when asking patients whether religion is important to them or not.
(2) Therapists’ concerns about bringing religion into therapy can be addressed through self-reflection, discussions with supervisors, and addressing discomfort rather than avoiding it.
(3) By adopting a respectful and curious position, therapists and patients can work together to explore the personal meaning and value of religious beliefs
